# Targeted Cytotoxic Therapy Kills Persisting HIV Infected Cells During ART

**DOI:** 10.1371/journal.ppat.1003872

**Published:** 2014-01-09

**Authors:** Paul W. Denton, Julie M. Long, Stephen W. Wietgrefe, Craig Sykes, Rae Ann Spagnuolo, Olivia D. Snyder, Katherine Perkey, Nancie M. Archin, Shailesh K. Choudhary, Kuo Yang, Michael G. Hudgens, Ira Pastan, Ashley T. Haase, Angela D. Kashuba, Edward A. Berger, David M. Margolis, J. Victor Garcia

**Affiliations:** 1 Division of Infectious Diseases, Department of Medicine, UNC Center for AIDS Research, University of North Carolina School of Medicine, Chapel Hill, North Carolina, United States of America; 2 Department of Microbiology, University of Minnesota, Minneapolis, Minnesota, United States of America; 3 Division of Pharmacotherapy and Experimental Therapeutics, UNC Eshelman School of Pharmacy, UNC Center for AIDS Research, University of North Carolina School of Medicine, Chapel Hill, North Carolina, United States of America; 4 Department of Biostatistics, UNC Center for AIDS Research, University of North Carolina School of Medicine, Chapel Hill, North Carolina, United States of America; 5 Laboratory of Molecular Biology, National Cancer Institute, National Institutes of Health, Bethesda, Maryland, United States of America; 6 Laboratory of Viral Diseases, National Institute of Allergy and Infectious Diseases, National Institutes of Health, Bethesda, Maryland, United States of America; National Institute of Allergy and Infectious Diseases, National Institutes of Health, United States of America

## Abstract

Antiretroviral therapy (ART) can reduce HIV levels in plasma to undetectable levels, but rather little is known about the effects of ART outside of the peripheral blood regarding persistent virus production in tissue reservoirs. Understanding the dynamics of ART-induced reductions in viral RNA (vRNA) levels throughout the body is important for the development of strategies to eradicate infectious HIV from patients. Essential to a successful eradication therapy is a component capable of killing persisting HIV infected cells during ART. Therefore, we determined the *in vivo* efficacy of a targeted cytotoxic therapy to kill infected cells that persist despite long-term ART. For this purpose, we first characterized the impact of ART on HIV RNA levels in multiple organs of bone marrow-liver-thymus (BLT) humanized mice and found that antiretroviral drug penetration and activity was sufficient to reduce, but not eliminate, HIV production in each tissue tested. For targeted cytotoxic killing of these persistent vRNA^+^ cells, we treated BLT mice undergoing ART with an HIV-specific immunotoxin. We found that compared to ART alone, this agent profoundly depleted productively infected cells systemically. These results offer proof-of-concept that targeted cytotoxic therapies can be effective components of HIV eradication strategies.

## Author Summary

Antiretroviral therapy (ART) improves the quality of life for HIV infected individuals. However, ART is currently a lifelong commitment because HIV persists during treatment despite being suppressed below detection. If therapy is stopped, the HIV reappears. A concerted effort is ongoing to develop new eradication therapies to prevent virus rebound, but there are challenges to be overcome. Our work is a major step forward in this process. We measured persistent HIV throughout the body during ART using bone marrow/liver/thymus (BLT) humanized mice, a model validated to study HIV persistence. HIV infected BLT mice were treated with tenofovir, emtricitabine and raltegravir. Despite documented tissue penetration by these drugs, we found that HIV expression persists in cells isolated from all the tissues analyzed (bone marrow, thymus, spleen, lymph nodes, liver, lung, intestines and peripheral blood cells). We therefore complemented ART with an immunotoxin that specifically kills HIV expressing cells while leaving other cells untouched. Our results demonstrate a dramatic reduction in persistent HIV throughout the body resulting from the killing of virus producing cells. Thus, our study provides new insights into the locations of HIV persistence during ART and a demonstration that persistent HIV can be successfully targeted inside the body.

## Introduction

ART is a lifesaving and effective means to control HIV infection [1]. However, the persistent nature of this infection requires lifelong adherence to daily ART dosing [2]–[4]. This viral persistence, the cumulative costs of ART, adverse events associated with long-term ART and the constant threat of emergence of drug-resistant viral variants have led researchers to pursue HIV eradication therapies that will result in a viral rebound-free interruption of therapy [2]–[4]. Towards this goal, “kick and kill” HIV eradication strategies are being developed [5]. While interventions that can induce expression of latent HIV (e.g. histone deacetylase inhibitors and protein kinase activators) will function as the “kick” [2]–[4], it is important to note that “kill” strategies cannot rely on the induction of virus expression in latently infected cells to result in cell death [6]. Therefore, candidate “kill” agents, such as immunotoxins, are being considered for incorporation into HIV eradication protocols [7], [8]. Immunotoxins are recombinant or biochemically linked bi-functional proteins that combine the effector domain of a protein toxin with the targeting specificity of an antibody or ligand [8]–[13]. Soon after HIV was identified as the causative agent of AIDS, several immunotoxins were described as potential therapeutics for HIV [8], [10]. These interventions had effector domains from plant and bacterial protein toxins and targeting moieties against either the HIV Env glycoprotein (gp120, gp41) or cellular markers including CD4, CD25 or CD45RO [14]–[21]. The immunotoxin we chose to evaluate for *in vivo* efficacy herein, 3B3-PE38, combines the 3B3 scFv which targets the conserved CD4 binding site of HIV-1 gp120 with the *Pseudomonas* exotoxin A (PE38) effector domain [22].

The fact that tissue specific effects of ART on HIV persistence are poorly understood in patients [23] meant that there was no baseline for characterizing the systemic effects of an immunotoxin on HIV persistence during ART. Therefore, it was essential that we first fully characterize the systemic impact of ART on HIV persistence cells *in vivo*. To do so, we sought to analyze HIV persistence during ART in a comprehensive panel of tissues and organs over time – a study that cannot be performed in human subjects. For this reason, we used BLT humanized mice [24], the most advanced, validated, and robust small animal model available for this purpose [25], [26]. The process of bioengineering BLT mice results in systemic dissemination of human hematopoietic cells throughout the animal [24], [27]. This phenotype facilitates the simultaneous analysis of multiple tissues throughout the body. The systemic effects of HIV infection on the BLT mouse human immune system (e.g., CD4^+^ T cell depletion and immune activation) recapitulate what is observed in HIV-infected patients [28]–[32]. Once the tissue-specific parameters of HIV persistence during ART were established, we incorporated 3B3-PE38 into the therapeutic regimen. Systemic analyses revealed that 3B3-PE38 treatment during ART reduced the number of HIV RNA producing cells to levels significantly lower than those achieved with ART alone. This observation demonstrates that immunotoxins can play a critical role in successful HIV eradication strategies.

## Results

### Outcomes of ART in HIV infected BLT mice

For this study, BLT mice were treated with a triple combination antiretroviral drug regimen that included the nucleotide reverse transcriptase inhibitor tenofovir disoproxil fumarate (TDF), the nucleoside reverse transcriptase inhibitor emtricitabine (FTC) and the integrase inhibitor raltegravir (RAL). This ART regimen was chosen because of its robust pharmacodynamic properties [33], superior efficacy in humans [34] and its efficacy in BLT mice [35].

**Figure 1:**
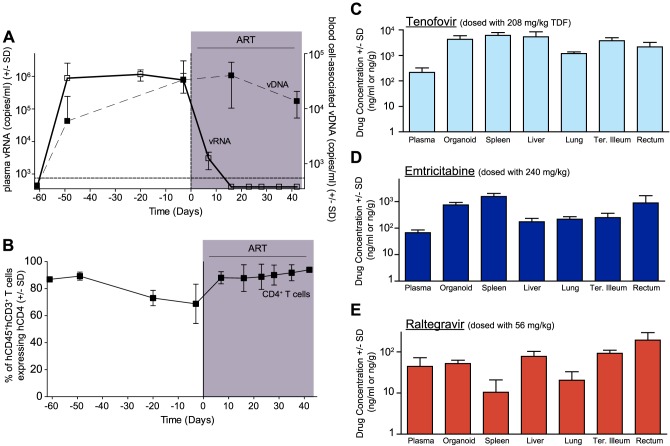
Efficacious plasma and tissue drug concentrations indicate broad dissemination of antiretrovirals during ART. (**A**) ART suppressed plasma viremia to below detection (vRNA; solid line, closed symbols) while peripheral blood cell-associated vDNA (dashed line; open symbols) were unaffected (Day −3 vs. Day42, p = 0.63, signed rank test). (**B**) ART led to a rebound in peripheral blood CD4^+^ T cells to pre-infection levels. (**C–E**) Drug concentrations [tenofovir (C), emtricitabine (D) or raltegravir (E)] were determined within the plasma and each of the indicated tissues. For this steady state drug concentration analysis, the 4 BLT mice in (A) were harvested 21 hours following their last ART dosing and the tissue concentrations of the antiretrovirals were determined.

In human peripheral blood [36], [37] and BLT mice (Fig. 1A), the first few weeks of ART are characterized by a rapid decline in plasma viremia (vRNA) followed by a plateau phase concomitant with a recovery of peripheral blood CD4^+^ T cells levels (Fig. 1B). We also observed that the blood cell-associated vDNA levels remained stable when compared to plasma vRNA levels during this treatment period (Fig. 1A) (Day −3 vs. Day42, p = 0.63, signed rank test), as seen in patients on this same ART regimen [38]. Furthermore, the robust suppression of plasma viremia by ART is consistent with the presence of each of the dosed antiretrovirals in the plasma of treated BLT mice: tenofovir (Fig. 1C), emtricitabine (Fig. 1D) and raltegravir (Fig. 1E). Such peripheral blood analyses are readily performed in both humans and BLT mice; however, the ability to simultaneously examine multiple tissues throughout the course of ART is not possible in patients. Therefore, we evaluated antiretroviral drug penetration as well as the impact of ART on vRNA production in multiple organs in BLT mice. We determined drug levels in the thymic organoid, spleen, liver, lung, terminal ileum and rectum of BLT mice. Importantly, each of the three drugs was detected in each matrix analyzed (Fig. 1C–E). To facilitate direct comparisons of drug levels between tissues and plasma, the data are presented as ng/g and ng/ml, respectively [39]. Overall, the tenofovir, FTC and RAL levels in BLT mice were ∼1.6 µM, ∼0.4 µM and ∼0.05 µM, respectively (Fig. 1C–E). Each of these values is higher than the EC_50_ for that drug in HIV-1 infected peripheral blood mononuclear cells (TDF: 0.005 µM; FTC: 0.01 µM; and RAL: 0.001 µM) [39]–[41]. The extensive antiretroviral drug penetration observed in BLT mouse organs during ART led us to examine the systemic impact of ART on HIV production.

**Figure 2:**
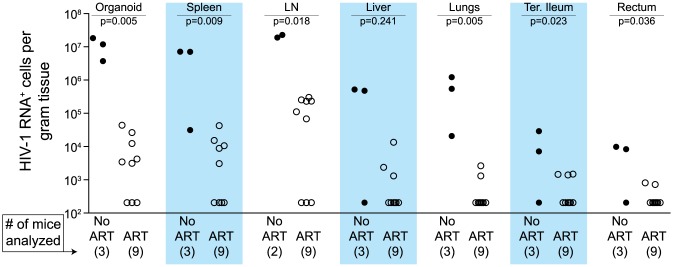
Durable reduction of the number of vRNA^+^ cells occurs during ART. Quantitative ISH analysis reveals statistically significant reductions in the numbers of productively infected cells for each tissue obtained from animals undergoing antiretroviral therapy. Exact log rank tests were utilized to generate p values. When no RNA^+^ cells were detected, then the number of RNA producing cells per gram tissue was set to 200 in the graph. (Closed symbols = no ART; open symbols = ART).

**Figure 3:**
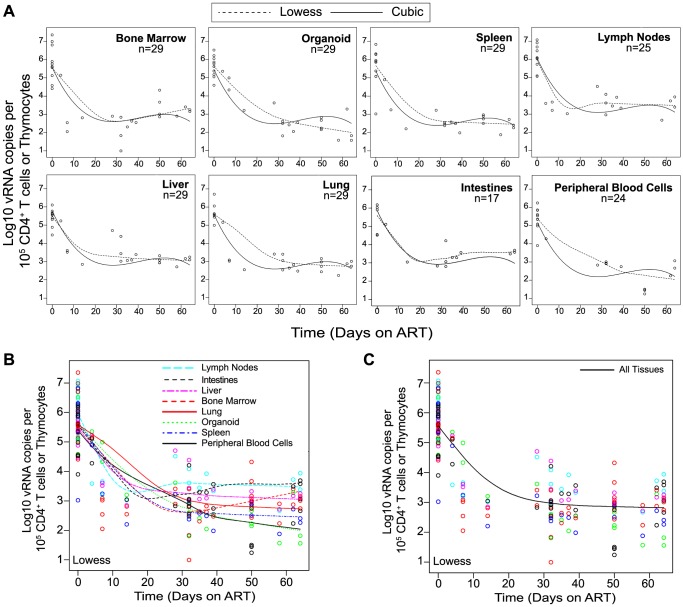
Viral RNA production during ART rapidly declines and then enters a plateau phase in all tissues. (**A**) Each data point indicates cell-associated vRNA levels (y-axis) and the days following ART initiation (x-axis) for a given BLT mouse in the indicated tissues. Both Lowess (dashed line) and a cubic (solid line) regression models were fit to the data for each tissue. (**B**) To facilitate comparative analyses between tissues, all of the Lowess regressions are graphed together. (**C**) To determine the overall impact of ART on systemic cell-associated vRNA levels, data from all of the tissues was used to generate a single Lowess curve.

**Figure 4:**
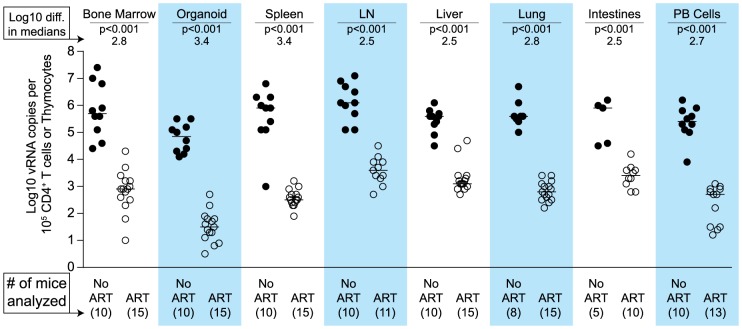
Each tissue analyzed exhibited a significant decline in vRNA during ART. When cell-associated vRNA levels during the plateau stage (treated 28–64 days) are compared to those from untreated mice, the reduction in vRNA levels were significant in all tissues (p<0.001). Reductions in cell-associated vRNA levels are presented as log10 differences in medians. Mann-Whitney tests were used to generate p values. (Closed symbols = no ART; open symbols = ART).

We used two different approaches to measure the systemic reduction in vRNA by ART. First, we used *in situ* hybridization to quantitate the number of individual cells producing vRNA in the human thymic organoid, spleen, lymph nodes, liver, lung, terminal ileum and rectum of HIV infected BLT mice receiving or not receiving ART. We found that antiretroviral penetration and activity in these tissues was sufficient to profoundly reduce the number of cells producing vRNA in all tissues (Fig. 2). However, consistent with the limited human tissue data available [42], vRNA producing cells remained detectable during therapy in all tissues analyzed (Fig. 2). We also used RT-PCR to quantitate cell-associated vRNA levels in the bone marrow, human thymic organoid, spleen, lymph nodes, liver lung, intestines and peripheral blood cells of BLT mice given ART for 0–64 days. We observed a rapid decline in cell-associated vRNA levels that plateaued by Day 28 of ART in all tissues analyzed (Fig. 3A). The longitudinal vRNA data was analyzed using two different regression models (Lowess and cubic). The observed reductions in cell-associated vRNA in each tissue tested indicate that ART penetrates these tissues sufficiently to significantly reduce viremia in a related manner in each tissue as illustrated in the plots depicting the individual Lowess curves graphed together (Fig. 3B). We found that a Lowess curve generated with cell-associated vRNA data for all tissues together (Fig. 3C) is very similar to the reduction in plasma viremia observed specifically in human peripheral blood soon after ART initiation [36]. We also noted significant (p<0.001) differences in each tissue, including peripheral blood, when we compared cell-associated vRNA levels from mice in an untreated state versus those from treated mice with vRNA levels within the plateau stage (Fig. 4). These similarities in vRNA reduction observed between human peripheral blood and all of the examined tissues from BLT mice during the first months following ART initiation suggest that blood is a reasonable surrogate for the impact of ART throughout the body.

### Impact of HIV-specific immunotoxin on residual vRNA production during ART

**Figure 5:**
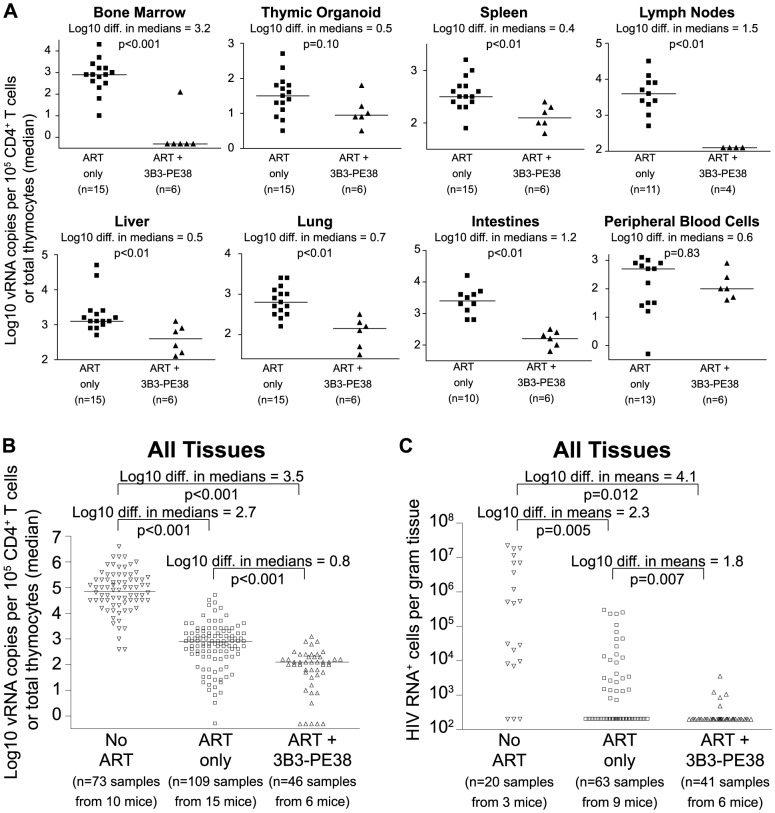
3B3-PE38 targets and systemically depletes vRNA^+^ cells *in vivo*. Beginning on Day 28 after ART initiation 3B3-PE38 was added to the treatment regimen every other day (7 total doses: 4 at 1 µg/25 g followed by 3 at 5 µg/25 g). The ART only control includes mice treated for 28–64 days. (**A**) Reductions in cell-associated vRNA levels are presented as log10 differences in medians. Mann-Whitney tests were used to generate p values. (**B**) Reductions in cell-associated vRNA levels for all tissues in (A) are graphed alongside data from untreated mice (Wilcoxon rank-sum statistics with repeated measures corrections). (**C**) Quantitative ISH for No ART mice (Fig. 2), ART only mice (Fig. 2) and the ART+3B3-PE38 group revealed reductions in the total number of HIV RNA producing cells per gram. When no RNA^+^ cells were detected, then the number of RNA producing cells per gram tissue was set to 200 in the graph (Exact log rank tests with repeated measures corrections).

Since neither cytopathic effects of HIV expression or antiviral immune responses are sufficient to deplete cells expressing virus [6], these cells represent potential targets for cytotoxic HIV-specific immunotherapies such as the immunotoxin 3B3-PE38. This immunotoxin has been shown to be active against HIV-1-infected primary CD4^+^ T cells, macrophages and thymocytes [43], [44]. Knowing that the effect of ART on the vRNA production reached a plateau by Day 28 of treatment led us to ask whether targeted killing of the persisting HIV producing cells during this plateau phase would augment the vRNA decline during ART [8]. As with most anti-HIV agents, monotherapy with immunotoxins is not clinically viable [8]; therefore, we quantitated the systemic impact of 3B3-PE38 on HIV persistence during ART (7 IP doses on alternate days beginning on Day 28 of ART). Compared to ART alone, ART plus 3B3-PE38 reduced the cell-associated vRNA by 3.2 logs (>1,000-fold) in the bone marrow (Fig. 5A). Complementing ART with 3B3-PE38 also led to a reduction in the persisting cell-associated vRNA levels in the human thymic organoid, spleen, lymph nodes, liver lung, intestines and peripheral blood cells (range: 0.4 to 1.5 logs) (Fig. 5A). When all tissues were analyzed together, ART plus 3B3-PE38 reduced cell-associated vRNA levels in all organs combined by an additional 0.8 logs compared to ART alone (p<0.001) (Fig. 5B).

Following the initiation of ART in humans, there is a multi-phasic decay in viremia that reflects the rate of turnover of productively infected cells [36], [45]. Most models of viral dynamics assume that the drugs completely block new infections of susceptible cells, with the observed decay reflecting the decay rate of cells infected prior to the initiation of ART [36], [46]. Because this decay is constantly progressing, it is likely that the number of vRNA producing cells would decline over time during ART in the absence of immunotoxin treatment. However, given that we observed an overall 0.8 log reduction in cell-associated vRNA levels beyond the effect of ART alone (Fig. 5B), we used *in situ* hybridization to examine whether there was an associated reduction in the number of cells producing vRNA. We found that complementation of ART with 3B3-PE38 led to a 1.8 log reduction in the numbers of vRNA producing cells throughout the body (p = 0.007) (Fig. 5C). This profound reduction in the number of vRNA producing cells provides a mechanistic explanation for the rapid reduction in tissue cell-associated vRNA levels relative to the ART only experimental group.

## Discussion

Despite the lifesaving benefits of ART in HIV patients, relatively little is known regarding the organ specific impact of this therapy on HIV production and persistence [23]. Improving our knowledge of the systemic effects of ART is a critical step in the development of successful HIV eradication therapies. To address this need, we characterized the impact of ART by analyzing both drug penetration and HIV production in different tissues throughout BLT humanized mice. We found that ART penetration into multiple organ systems is sufficient to significantly reduce the number of cells producing HIV, as well as cell-associated vRNA levels, throughout the body. However, HIV persists during ART in every organ analyzed.

**Figure 6:**
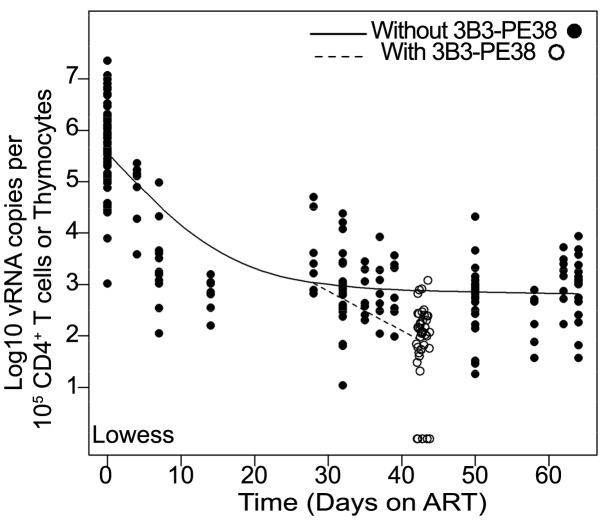
The 3B3-PE38 mediated killing of vRNA producing cells leads to a more rapid reduction in vRNA levels versus ART only. The single Lowess curve for all data points in Fig. 3C (closed symbols; solid line) is graphed together with the combined tissue data for ART+3B3-PE38 (open symbols; dashed line) to reveal the alteration in cell-associated vRNA levels over time due to the immunotoxin. The beginning of the plateau phase of decay (Day 28) is the divergence point.

HIV-infected cells persisting during ART represent suitable targets for cytotoxic HIV-specific immunotherapies such as the 3B3-PE38 immunotoxin. Since such therapies target HIV proteins expressed by infected cells, their efficacy requires active virus transcription. Therefore there is no predicted impact of immunotoxin treatment on the size of the transcriptionally silent latent HIV reservoir. For this reason, the HIV latent reservoir was not quantitated in this study and our conclusions are based on the quantitation of cell-associated vRNA and vRNA-producing cell numbers. Specifically, we demonstrate that 3B3-PE38 kills vRNA producing cells throughout the body such that the reduction of vRNA levels during combined therapy is more rapid than with ART only (Fig. 6). A recent report described the ability of new broadly neutralizing monoclonal antibodies to suppress HIV-1 rebound after termination of ART [47] which led us to consider the possibility that the observed activity of the immunotoxin was due in part to neutralization by the 3B3 scFv moiety. In the Horwitz, et al. study, the amount of IgG (10.5 mg/injection) administered was over 2000 fold higher than our doses of 3B3-PE38 (0.005 mg/injection). Thus, the circulating 3B3-PE38 levels in our study could not reach the levels required for neutralization by 3B3 [48]. It is therefore unlikely under our experimental conditions that neutralization by the 3B3 scFv moiety of the immunotoxin accounts for the significant 3B3-PE38-mediated reduction we observed in tissue cell-associated vRNA levels.

Comparing and contrasting data from BLT mice with data from patients and NHP is essential to understanding the predictive nature of our model. The most notable difference between our study and those in patients and NHP is the duration of ART. Our study examined the impact of ART in BLT mice over ∼2 months, while patient and macaque studies have examined cell-associated vRNA levels during several years of ART [49], [50]. These differences in ART duration prevent direct comparisons of the impact of long-term ART on persistent vRNA production within these experimental platforms. Despite this constraint, we can compare data across all three experimental platforms for consistency. For example, we found that our data showing the continued presence vRNA in BLT mouse peripheral blood and tissue cells during ART are consistent with the continued presence of vRNA within patient peripheral blood cells, ileal biopsy cells and rectal biopsy cells from patients treated for a median of 12.5 months with ART [50] and within macaque cells from throughout the body after 26 weeks on ART [49]. In addition to future studies incorporating longer treatment windows in BLT mice, it will be important to determine whether the vRNA plateau phase reached after 28 days is directly comparable to that seen in humans on long term ART. For this purpose, incorporation of an additional drug into the ART regimen could be tested. If the plateau state in BLT mice is comparable to the human situation during long-term ART, then future studies using more sensitive assay for HIV-1 RNA in conjunction with ART intensification should not detect any additional reduction in the steady state level of viremia during this timeframe.

The results presented here provide proof of concept for targeted cytotoxic therapy as a successful complement to ART for the depletion of persisting HIV infected cells. In addition to the 3B3-PE38, alternative immunotoxins with different effector domains and targeting moieties are available [8]–[10]. This is important because: (i) treatments with different cytotoxic interventions during ART will likely be required to completely eradicate HIV producing cells due to the immunogenicity of the immunotoxin molecules [8]–[13], [51], [52], the need to target the diverse cell types that are productively infected with HIV [2]–[4], and (iii) the likely selection of viral mutants resistant to any single immunotoxin [53]. Beyond immunotoxins, alternative targeted cytotoxic therapy strategies developed for cancer treatment could also be adapted to generate comprehensive anti-HIV cytotoxic therapy regimens [54], [55]. Possibilities include: aptamer—toxin conjugates; radioimmunotherapies; antibody—cytotoxic drug conjugates; targeted cytolytic viruses; targeted delivery of a cytotoxic peptides; and adoptive immunotherapy with *ex vivo* expanded natural or genetically modified T cells [54], [55]. Importantly, the model system developed herein to quantitate the effects of 3B3-PE38 can be used to determine the *in vivo* efficacy of any of these other strategies alone or in combinations.

In summary, we determined that the systemic penetration of antiretroviral drugs into different organs throughout the body was sufficient to reduce cell-associated vRNA levels ART alone was unable to completely eliminate vRNA expressing cells in tissues. This observation provided a quantitative framework for the systemic *in vivo* efficacy evaluation of interventions to destroy HIV producing cells during therapy. Within this framework we were able to demonstrate that persistent HIV producing cells systemically present during ART were significantly depleted by an Env-targeting immunotoxin. Such targeted cytotoxic interventions may prove to be critically important components of an effective HIV eradication strategy.

## Materials and Methods

### Ethics statement

All animal experiments were conducted following NIH guidelines for housing and care of laboratory animals and in accordance with The University of North Carolina at Chapel Hill (UNC Chapel Hill) regulations after review and approval by the UNC Chapel Hill Institutional Animal Care and Use Committee (permit number 12-171).

### Generation of BLT humanized mice

BLT mice were prepared essentially as previously described [27]. Briefly, thy/liv implanted NOD/SCID-gamma chain null mice (NSG; The Jackson Laboratories, Stock #5557) were transplanted with autologous human fetal liver CD34^+^ cells (Advanced Bioscience Resources) and monitored for human reconstitution in peripheral blood by flow cytometry [24], [27], [32], [35]. All BLT mice (n =  40) used for these experiments were characterized for human immune system reconstitution prior to HIV infection. Their peripheral blood contained an average of 57% (^+^/−15 SD) human CD45^+^ cells of which 58% (^+^/−22 SD) were human T cells. Of the human T cells, 81% (^+^/−6 SD) were human CD4^+^ T cells.

### Analysis of HIV infection of BLT mice

Infection of BLT mice with HIV-1_JRCSF_ was monitored in peripheral blood by determining plasma levels of vRNA as described (level of detection = 750 copies per mL of plasma; 40 µl mouse plasma sample size) using one-step reverse transcriptase real-time PCR [ABI custom TaqMan Assays-by-Design] [35], [56]–[58]. Tissues were harvested and cells isolated as we have previously described for RNA isolation or flow cytometric analysis [24], [32]. Flow cytometry data were collected using a BD FACSCanto cytometer and analyzed using BD FACSDiva software (v. 6.1.3).

For HIV RNA *in situ* hybridization (ISH), tissues were fixed overnight in 4% paraformaldehyde at 4°C and then transferred to 70% ethanol. Tissues were then embedded in paraffin for sectioning. After deparaffinization with xylene and rehydration through graded ethanols, tissue sections were treated with HCl, triethanolamine, digitonin and 4 µg/mL Proteinase K as previously described [59]. After acetylation with acetic anhydride and dehydration, tissue sections were hybridized at 45°C overnight with a ^35^S labeled antisense riboprobe and 0.5 mM aurintricarboxylic acid in the hybridization mix. After extensive washes and ribonuclease treatment, tissue sections were dehydrated, coated in Ilford K5 emulsion diluted with glycerol and ammonium acetate, exposed at 4°C for 7–14 days, and developed and fixed per manufacturer's instructions. They were stained with Hematoxylin, dehydrated, and mounted with Permount. Non-infected control tissues and sense riboprobe controls were analyzed when appropriate. Photographic images using epifluorescence were taken with a digital camera and the tiffs were analyzed for the area of the sections and the area occupied by silver grains using Photoshop with Fovea Pro. Section weights were estimated from their 5-micron thickness and their area. These methods have been extensively reviewed [59].

### Treatment of HIV infection of BLT mice

ART in infected BLT mice consisted of three antiretrovirals administered daily via intraperitoneal injection: tenofovir disoproxil fumarate (TDF; 208 mg/kg), emtricitabine (FTC; 240 mg/kg body weight) and raltegravir (RAL; 56 mg/kg) [35]. The gp120-targeting immunotoxin 3B3(Fv)-PE38 [22] (here referred to as 3B3-PE38) was administered intraperitoneally every other day beginning on Day 28 of ART for a total of 7 doses: the first 4 were 1 µg/25 g and the last 3 were 5 µg/25 g.

### Analytical methods for drug quantification

Plasma and snap frozen tissue samples from BLT mice receiving daily ART were analyzed for drug concentrations. Quantification of FTC, tenofovir (TFV) and RAL plasma concentrations was performed by protein precipitation and LC-MS/MS analysis. 10 µl of each stored plasma sample was mixed with 75 µL of methanol containing the isotopically-labeled internal standards (^13^C TFV and ^13^C ^15^N FTC). Following vortexing and centrifugation, the supernatant was removed and evaporated to dryness. The extracts were reconstituted with 1 mM ammonium phosphate prior to LC-MS/MS analysis. All compounds were eluted from a Phenomenex Synergi Polar-RP (4.6×50 mm, 4 µm particle size) analytical column. An API-5000 triple quadrupole mass spectrometer (AB Sciex, Foster City, CA) was used to detect the analytes. Data were collected using AB Sciex Analyst Chromatography Software (Analyst version 1.6.1). The dynamic range of this assay was 1–1000 ng/mL for each compound using a 1/concentration^2^ weighted linear regression. Calibrators and quality control samples were within 15% of the nominal value for all compounds. Plasma samples with concentrations above the calibration range were diluted into the calibration range with blank plasma prior to reanalysis.

The quantitation of FTC, TFV and RAL in tissues started with the homogenization of each tissue in a 70% acetonitrile + 30% 1 mM ammonium phosphate solution. A portion of the resulting homogenate was extracted by protein precipitation with acetonitrile containing isotopically-labeled internal standards (^13^C TFV, and ^13^C ^15^N FTC). Following vortexing and centrifugation, the supernatant was removed and evaporated to dryness. The extracts were reconstituted with 1 mM ammonium phosphate prior to LC-MS/MS analysis. TFV and FTC were eluted from a Waters Atlantis T3 (100×2.1 mm, 3 µm particle size) analytical column. RAL was eluted from a Phenomenex Synergi Polar-RP (50×4.6 mm, 4 µm particle size) analytical column. An API- 5000 triple quadrupole mass spectrometer was used to detect all analytes. Data were collected using AB Sciex Analyst Chromatography Software. The dynamic range of this assay was 0.3–300 ng/mL of homogenate for each compound using a 1/concentration^2^ weighted linear regression. Based upon the mass of the tissue extracted, the concentrations were converted from ng/mL homogenate to ng/g tissue. Calibrators and quality control samples were within 15% of the nominal value all compounds.

### Statistics

All statistical tests performed using an alpha level of 0.05. Exact log rank tests were utilized to generate p values for Fig. 2 (R v2.14.1). Mann-Whitney comparisons were utilized to generate the p values presented in Figs. 4 & 5A (Prism v4). Wilcoxon rank-sum statistics with repeated measures corrections were utilized to generate p values presented in Fig. 5B (R). Exact log rank tests with repeated measures corrections were utilized to generate p values for Fig. 5C (R). For Figs. 3 & 6, the Lowess and cubic regression models were fit using R. The linear heterogeneity models that allowed for the residual variance to change over time were computed in SAS/STAT software. Graphs were generated in Prism v4 with the exception of the fitted Lowess and cubic regression models which were generated in R.
